# The Art and Science of Securing an Anaesthesiology Training Job: A Survey of the Key Factors in Two Australian Tertiary Hospitals

**DOI:** 10.7759/cureus.58879

**Published:** 2024-04-23

**Authors:** Michael Sawang, Anne-Marie (Amie) Dempster, Steven Cai

**Affiliations:** 1 Department of Anaesthesiology, Prince of Wales Hospital, Sydney, AUS; 2 Medicine, University of New South Wales, Sydney, AUS

**Keywords:** anaesthesia, anzca, applicant selection, subspecialty training, residency recruitment

## Abstract

Background

There is no specific formal guidance on what prospective trainees must focus on to secure an anaesthetic training position in Australia, and there is little in the literature to advise both applicants and their mentors.

Method

This study aims to ascertain the views of anaesthetic clinicians from two Australian tertiary referral hospitals on what they consider most important for selection. A paper-based survey was conducted at both hospitals across three groups, totalling 104 participants with a 100% response rate.

Results

The characteristics most agreed upon to be of at least some importance were clinical anaesthetic knowledge (98%, 102/104), teaching (95%, 99/104), basic science and courses (94%, 98/104), other critical care experience (93%, 97/104), and anaesthetic experience for more than six months (92%, 96/104). Of these, anaesthetic experience of greater than six months, non-anaesthetic critical care experience, and the completion of relevant courses were felt to be most important. Furthermore, good referee reports (95%, 99/104), especially those that come from anaesthetists (75%, 78/104) as well as having previous experience working in the institution applied to (88%, 92/104) were also seen as important factors. ‘Non-technical’ skills (40%, 42/104) were also regarded as an important factor, with immense competition for a few training positions (45%, 47/104) as the greatest barrier. When it came to selection, prevocational trainees consistently ranked the majority of criteria higher than accredited trainees or specialists.

Conclusion

This staff survey in two Australian hospitals has shed light on factors considered critical in securing an anaesthetic training position. It underscores the significance of clinical anaesthetic knowledge, basic science proficiency, and relevant critical care experience.

## Introduction

Aspiring anaesthetic trainees face intense competition in securing an anaesthetic training position in Australia [1]. The Australian and New Zealand College of Anaesthetists (ANZCA) provides a guide to trainee selection based on the ANZCA Roles of Practice [2]. However, the crucial factors for selection remain unclear.

For prospective trainees, the absence of specific guidance on which area to focus on is discomforting. Many then turn to informal input from colleagues and senior clinicians to understand what is most important, despite the potential for individual biases and reliance on anecdotes. To our knowledge, there are no studies published in Australia that have explored the attitudes of anaesthetic clinicians towards what is required to gain an edge in selection.

To investigate this further, we performed a cross-sectional survey of three groups: prospective trainees, vocational trainees, and consultants, in two major tertiary referral centres, with the primary goal of ascertaining their views on what they believe the most important factors are for selection. As a secondary aim, we attempt to identify differences between the three groups. In doing so, we hope to provide better insight and direction for prospective trainees on what activities they should focus on to maximise their chances. 

## Materials and methods

Ethics

This project was submitted to the South-Eastern Sydney Local Health District (SESLHD) Research Ethics and Governance Office, which deemed that there were no ethical risks.

Participants and setting

The survey was undertaken at two tertiary referral hospitals in New South Wales: Prince of Wales Hospital, Sydney, and the Sydney Children's Hospital, Randwick. To ensure optimal response rates, we distributed the survey through paper-based questionnaires from April 14th, 2023, to May 15th, 2023. Surveys were distributed, and written responses were then collected individually an hour or so later. The forms were flipped over during collection and then placed in a large, covered box. Data was entered electronically and only reviewed upon completion of all surveys. It conforms to previously issued guidelines on survey research [3,4].

There were three sections to the survey: in part one, there were 12 questions examining the specific characteristics of trainees, with respondents detailing the degree of importance on a five-point Likert scale (Appendix 1).

In part two, there were ten statements exploring the impact of referees and factors relating to the selection process itself, to which participants rated the strength of their agreement or disagreement, also on a five-point Likert scale (Appendix 2).

In part three, there were three open-ended, free-text questions to ascertain views on the most important criteria for selection, the largest barriers, and potential avenues to improve the selection process (Appendix 3).

Survey questions were prepared in accordance with current ANZCA guidelines and through feedback from senior departmental staff. They underwent peer review and pilot testing by the Prince of Wales Hospital anaesthetic research group as well as two external anaesthetists.

Data analysis

Likert survey responses to the presented questions were treated as ordinal data and analysed descriptively, both in aggregate and then as subgroups. Due to the large number of potential comparisons, only five predetermined statistical tests were made between the subgroup responses.

Selection factors analysed were: anaesthetic experience of six months; basic science knowledge; further postgraduate education; courses; and research. The authors selected these questions because they believed them to be crucial factors.

Statistical analysis was conducted using IBM SPSS Statistics for Windows, Version 26 (released 2019; IBM Corp., Armonk, New York, United States). The Kruskal-Wallis H and Mann-Whitney U tests were used given the non-parametric nature of our dataset, and significance levels were set at p < 0.01 and p < 0.0033, respectively, after applying the Bonferroni correction for multiple comparisons.

The authors independently analysed responses to the open-ended questions and generated themes considered to best describe the data. Researchers checked and discussed the findings.

## Results

A total of 104 participant surveys were collected from Prince of Wales Hospital and the Sydney Children's Hospital, of which there was a 100% response rate. This included 15 prevocational trainees (14%), 32 accredited trainees (31%), and 57 consultants (55%).

**Figure 1:**
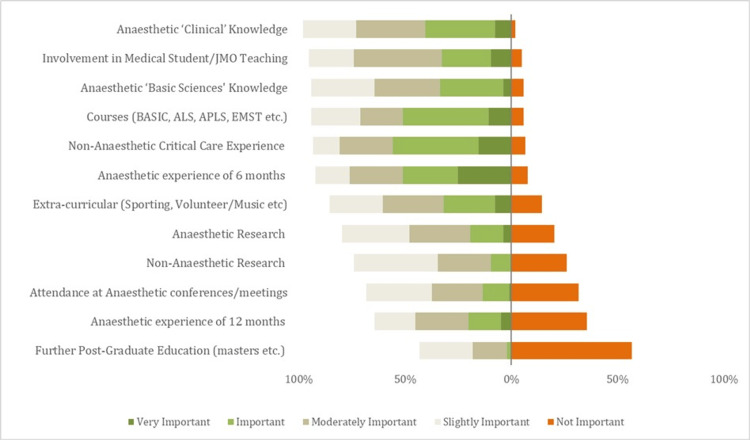
Converging stacked bar chart demonstrating aggregate results for part one of the survey Green (light and dark) represent 'important' and 'very important', respectively. Red represents ‘not important’. ‘Moderately important’ and ‘slightly important’ are represented by light-grey colouring, respectively. Attributes were ranked from top to bottom based on the percentage of responses that deemed attributes to be at least ‘slightly important’.

In part one of the survey, prospective trainee characteristics were assessed from ‘not important’ to 'very important', with these data represented in Figure 1. Here, attributes were ranked according to the frequency of responses describing them as of at least some importance.

The factors seen most frequently as important (slightly to very important) by respondents were clinical anaesthetic knowledge (98%, 102/104), teaching (95%, 99/104), basic sciences and courses (both 94%, 98/104), other critical care experience (93%, 97/104), and anaesthetic experience of more than six months (92%, 96/104).

Of these, the factors most rated as very important were an anaesthetic experience of six months (25%, 26/104), followed by non-anaesthetic critical care experience (15.4%, 16/104), and relevant courses (10.6%, 11/104). Of lesser importance were extracurricular activities (86%, 89/104) and research (both anaesthetic [80%, 83/104] and non-anaesthetic [74%, 77/104]).

Finally, attendance at anaesthetic conferences or meetings (68%, 71/104), the anaesthetic experience of 12 months (65%, 67/104), and pursuing post-graduate education such as a master's (43%, 45/104) were seen as relatively of least importance.

**Figure 2:**
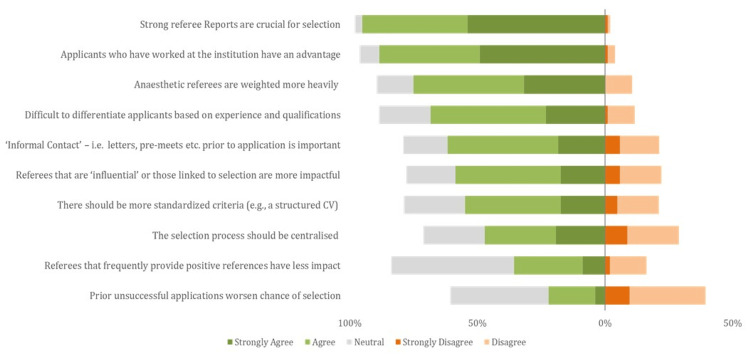
Converging stacked bar chart demonstrating aggregate results for part two of the survey Green (light and dark) represent ‘agree’ and ‘strongly agree', respectively. Red (light and dark) represents ‘disagree’ and 'strongly disagree‘, respectively. Neutral is represented by light-grey colouring. Questions were ranked from top to bottom based on the percentage of responses that were ‘strongly agree’ or ‘agree’.

In part two of the survey, statements pertaining to referees and the selection process were ranked by the proportion of responses that either ‘agreed’ or ‘strongly agreed’ (Figure 2). Most respondents believed that strong referee reports were crucial for selection (95%, 99/104), that familiarity with hospitals was advantageous (88%, 92/104), and that anaesthetic references were weighted more heavily than non-anaesthetic ones (75%, 78/104).

A substantial proportion of people also agreed that it was difficult to differentiate applicants based on experience and qualifications (68%, 71/104). More than half of respondents agreed that informal contact prior to application was important (62%, 64/104), that ‘influential’ referees were more impactful (59%, 61/104), and that selection criteria should be standardised (55%, 57/104). A slightly lower proportion agreed with the centralisation of the recruitment process (47%, 49/104). Even fewer agreed that referees who frequently provide positive references have less impact (36%, 37/104) and that prior unsuccessful applications worsen the chances of selection (22%, 23/104).

**Figure 3:**
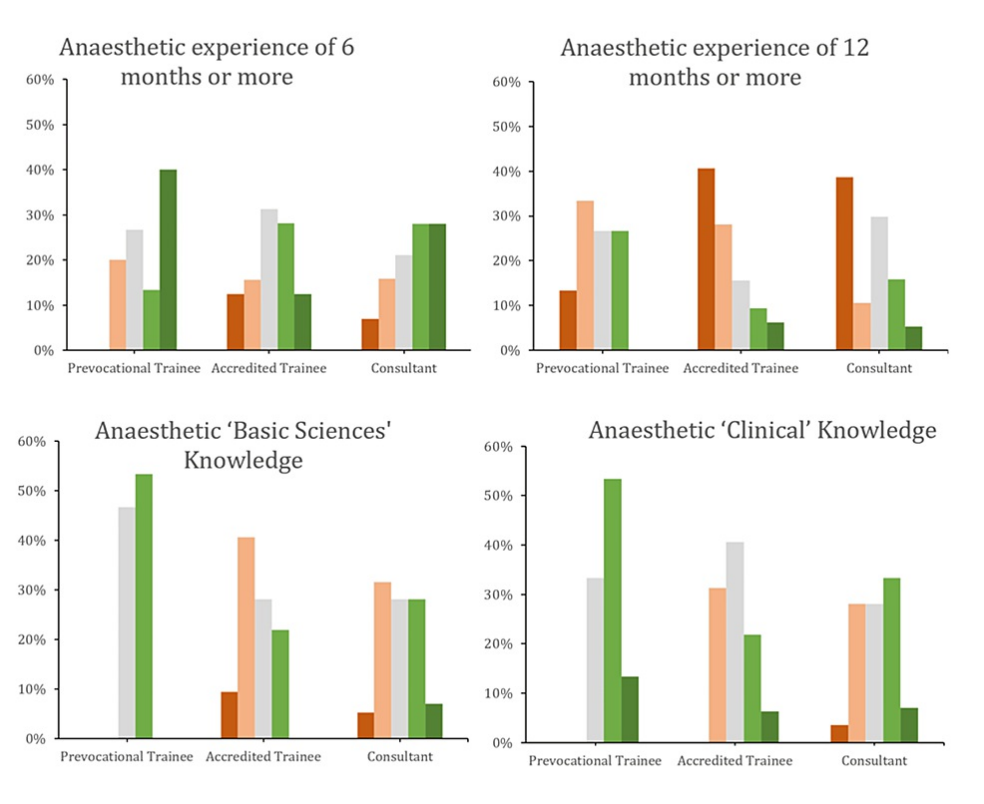
Question responses stratified by subgroup Green (light and dark) represent 'important' and 'very important', respectively. Red (light and dark) represent ‘slightly important’ and ‘not important', respectively. ‘Moderately important’ is represented by light-grey colouring.

**Figure 4:**
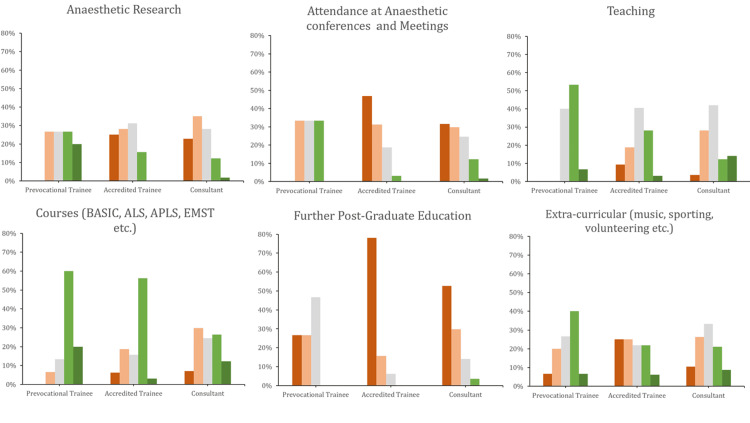
Question responses stratified by subgroup Green (light and dark) represent 'important' and 'very important', respectively. Red (light and dark) represent ‘slightly important’ and ‘not important’, respectively. ‘Moderately important’ is represented by light-grey colouring.

The responses from the prevocational trainees were significantly different from the other two groups. They attributed more importance than vocational trainees or specialists to most factors, including anaesthetic experience, clinical and basic sciences knowledge, courses, further education, research, and teaching, than did accredited trainees or specialists (Figures 3, 4). Furthermore, they were more likely to disagree with the statement that prior unsuccessful applications worsen the chances of selection and agree with the idea of standardised selection criteria.

**Figure 5:**
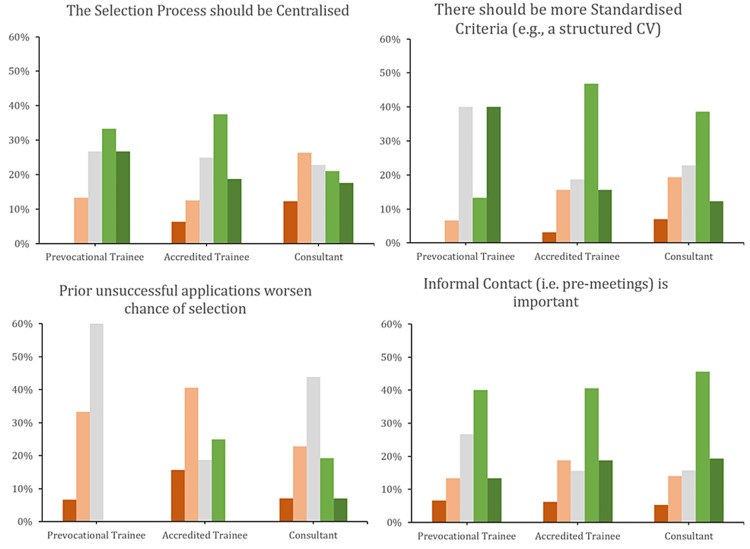
Question responses stratified by subgroup Green (light and dark) represent ‘agree’ and ‘strongly agree’, respectively. Red (light and dark) represent ‘disagree’ and ‘strongly disagree’, respectively. ‘Neutral’ is represented by light-grey colouring.

Interestingly, accredited trainees rated extracurricular activities as less important. A greater proportion of consultants disagreed with centralisation compared to the other two groups (Figure 5).

Statistical analysis

Our statistical analysis of the five key factors, determined a priori, broadly supported these observations. Regarding anaesthetic ‘basic sciences’ knowledge, there was a statistically significant difference between groups (P = 0.01), found to be between prevocational and accredited trainees. Similarly, a significant difference was found for anaesthetic research (P = 0.009), driven by a difference between prevocational trainees and consultants (P = 0.003). Specialists were significantly more likely to value research than prevocational trainees. Differences were also found for further education (P < 0.001) between prevocational and accredited trainees. There were no statistically significant differences between groups concerning the importance of courses or anaesthetic experience of six months or more.

The results of the open-ended questions are presented below. Some participants provided multiple criteria that they felt were most important. 

The most important factor for selection by responder frequency was ‘non-technical’ skills (40%). These responses referred to qualities such as being personable, pleasant, teachable, humble, and having good teamwork and communication skills. Other responses were similar in sentiment and included demonstrating commitment both to anaesthetic training as well as the department of interest (e.g., enthusiasm, audits, and research) (19%) and being known to the department of application (18%) as well as strong references (21%). Work ethic was nominated as the most important factor by 11% of respondents. Only 6% of people were unsure or did not answer.

The most common barrier that was nominated was the strong competition for the few training positions (45%). The second-most-cited hurdle was attaining the necessary anaesthetic experience to be competitive (23%). Other challenges included difficulty becoming known, insufficient time in anaesthesia, and a lack of clarity over selection criteria. There was also consternation about constantly being rotated to a different consultant on surgical lists and the concomitant difficulties in building familiarity. Thirteen participants (13%) either responded unsure or left the question blank.

The most common suggestion for improvement of the selection process was for a centralised recruitment process (37%) and more clarity over selection criteria (24%). A significant number of people were unsure or left the answer blank (29%). A few responses suggested increasing interview time, adding a simulation or objective structured clinical examination (OSCE), discouraging informal meetings, better evaluation of non-technical skills, feedback after failed applications, and additional pathways to increase the unaccredited anaesthetic time. 

## Discussion

The majority of research on the selection of anaesthetic trainees has focused on factors that predict success in exams/performance [5-7] or factors that attract or retain trainees [8,9]. None have evaluated, to our knowledge, which criteria are most important in securing a position.

Our study is the first in Australia to examine the attitude of anaesthetic clinicians towards the relative importance of various factors for selection into anaesthetic training. Additionally, we were also able to examine factors relating to referees and the selection process. 

In part one, the most important candidate characteristics identified are an anaesthetic experience of at least six months, followed by non-anaesthetic critical care experience and completion of appropriate courses. While the amount of anaesthetic experience that is desirable is unclear, it does appear there are diminishing returns once the threshold of six months is reached, especially given the relatively low importance ranking of 12 months of experience. Feedback within our survey suggests that non-anaesthetic critical care experience improves trainees' ability to manage critically ill patients and emergencies.

In part two, there was widespread agreement that strong referee reports and prior experience working at the same institution are important for selection. Perhaps reports from fellow anaesthetists are considered a more reliable predictor of an applicant’s suitability for anaesthetic training compared to other specialists. Referee reports are important as they are often used as a surrogate measure to assess cognitive and interpersonal skills, which are otherwise difficult to directly examine unless the candidate is well known to the institution of application.

There has been a stronger emphasis in anaesthesia on non-technical skills in recent decades [10]. Part three of this study supports this trend, where non-technical skills (communication, teamwork, diligence, trainability, situational awareness, etc.) were seen by respondents as the most important attributes for selection.

Applicants tend to select referees who will describe them most positively. This study supports the claim that being known or having worked at an institution is important. Perhaps this reflects the emphasis placed on direct observation as opposed to relying on surrogate reports. Another useful finding for prospective trainees from this study is that referees with a reputation for providing positive references are not regarded as being less impactful.

The survey also supports the idea that proficiency in the basic sciences is an important factor. Prior to 2013, many aspiring trainees were advised that a critical step in distinguishing themselves was to succeed in the primary exam. This is far less feasible now, as since 2013, only accredited trainees are permitted to sit for the primary exam. Successful completion of the basic science exams, such as the anaesthetic primary exam, has invariably been viewed favourably. Indeed, several studies in the US have linked United States Medical Licencing Examination (USMLE) scores [5,6,11] with subsequent performance in anaesthesiology residency.

Overall, further post-graduate education, research, and attendance at anaesthetic conferences were not felt to be very important. Prevocational trainees assigned more importance to these attributes than vocational trainees or consultants did. These differences were statistically significant. One orthopaedic paper from the US also found that there were important differences in the perceptions of faculty and residents in what they gauged as the most important attributes to succeed in obtaining traineeships [12].

Our findings of these discrepancies between prevocational trainees and the other two groups possibly reflect a disconnect in understanding the process. We hope that the current study will help to assist those prevocational trainees in determining what matters most. A recent interview-based study in Australia strongly corroborates these findings [13]. They discovered a key barrier to attaining speciality training was navigating a complex and unpredictable system with minimal guidance. They also attest to the heavy pressure that candidates face, which is also reflected in the present study, where most respondents reported overwhelming competition for limited positions.

The survey was conducted in New South Wales, a state in which specific hospital networks conduct their own processes and reviews of applicants. Some Australian states have centralised systems for trainee selection. In the state of Victoria, for instance, the Victorian Anaesthesia Training Scheme (VATS) oversees the recruitment process. The criteria used in VATS mirror those of ANZCA and also lack specific details that could guide aspiring anaesthetists. A working party has been convened by the Australian and New Zealand College of Anaesthetists to improve clarity in selection criteria and consistency across training networks [1].

In our study, we found that respondents cited recruitment centralisation most frequently when asked about potential improvements to the selection process. Many respondents supported standardisation of criteria (with a structured Curriculum Vitae (CV) proposed as an example in the question wording); we speculate that this may address some of the misconceptions of prevocational trainees. Additionally, centralisation of selection may address the barriers within NSW to applicants who may not be known or familiar with the network to which they are applying and allow more equal applications everywhere. The difficulty faced by applicants in ‘breaking in’ to a network is strengthened by our finding that a substantial proportion of respondents alluded to being known/having worked with the institution of application as being crucial. Centralisation may also help address the currently burdensome application process required for each individual hospital/network.

While more people tended to agree than disagree with the idea of centralisation of selection, the views of our cohort were varied, especially amongst consultants. The current system is advantageous in the ability of individual departments to exert control over selection. They are uniquely able to directly observe many of their candidates prior to application and scrutinise their own hiring practices, and they may be more judicious in finding candidates whose career goals and personalities are more aligned.

Limitations

Our study has several limitations worth noting. While we achieved a 100% response rate, our survey was confined to two hospitals in Sydney. However, many of these professionals also work in other hospitals across New South Wales (NSW). Specifically, more than 95% of the specialists and all vocational trainees practice elsewhere, indicating that the views captured extend beyond the two surveyed hospitals.

Another concern is the potential for bias in paper surveys. While we initially considered an email approach for anonymity and blinding, we ultimately opted for face-to-face, paper-based surveys to enhance response rates. To improve concealment, surveys were collected with the written side flipped over and only recorded electronically after completion.

Notably, this survey does not consider written responses to job application questions or interview performance, which are both key factors. The relative importance of individual selection criteria may also vary depending on the stage of the process; criteria for selection may differ between the initial selection stage and the interview stage. Additionally, within a hospital setting, emphasis on certain factors may shift, such as considerations for the mix of trainees or temporary position coverage. It is also important to acknowledge that the priorities of a small number of consultants who wield direct influence in selection may differ from those of their specialist colleagues.

As the study was geographically limited to NSW, our questions may be less applicable to other states. Future research in other Australian states with centralised selection may be useful to delineate possible differences.

## Conclusions

This survey of anaesthetic staff at two Australian tertiary hospitals offers insight into the key factors influencing the selection of anaesthetic trainees. These findings could be beneficial to aspiring trainees and the specialists guiding them in their career paths.

## Appendices

Appendix 1

**Table 1:** Survey: part one Participants were asked to rate each statement as “not important”, “slightly important”, “moderately important”, “important” or “very important”.

Experience and proficiency:
1. Anaesthetic experience of 6 months or more
2. Anaesthetic experience of 12 months or more
3. Anaesthetic ‘basic sciences’ knowledge
4. Anaesthetic ‘clinical’ knowledge
5. Non-anaesthetic critical care experience (PGY 3 and above)
Education/Courses:
1. Further post-graduate education (i.e. master's or PhD) completed or in progress
2. Courses (BASIC, ALS, APLS, EMST, CICO, EMAC, etc.)
3. Attendance at anaesthetic conferences and meetings
Research:
1. Anaesthetic research (ongoing or published) including audits/presentations
2. Non-anaesthetic research (ongoing or published) including audits/presentations
Teaching:
1. Involvement in medical student/junior doctor teaching
Personal characteristics:
1. Extra-curricular (sporting, art, volunteer/music, etc.)

Appendix 2

**Table 2:** Survey: part two Participants were asked to rate each statement on a scale of 1 to 5 with possible responses being “strongly disagree”, “disagree”, “neither agree nor disagree”, “agree” and “strongly agree”.

Referees:
Strong referee reports are crucial for selection
Anaesthetic referees are weighted more heavily than non-anaesthetic referees
Referees that are ‘influential’, ‘well-known’ persons or those linked to selection are more impactful
Referees who frequently provide positive references have less impact
Selection process:
‘Informal contact’ i.e. phone calls, emails, letters, pre-meets, etc. before the application is important
Applicants who have previously worked at the institution they are applying for have an advantage
Prior unsuccessful applications worsen the chance of selection
The selection process should be centralised by the state
There should be more standardized criteria (e.g., a structured Curriculum Vitae (CV)) for prospective applicants
It is difficult to objectively differentiate applicants based on experience and qualifications

Appendix 3 

**Table 3:** Survey: part three

Short answer questions:
1. What do you feel is the most important selection criteria and why?
2. What are the biggest barriers to getting onto the training program?
3. Do you feel that anything could be improved and if so how?
